# Inhibition of Cytosolic Phospholipase A_2_α (cPLA_2_α) by Medicinal Plants in Relation to Their Phenolic Content

**DOI:** 10.3390/molecules200815033

**Published:** 2015-08-17

**Authors:** Eva Arnold, Thorsten Benz, Cornelia Zapp, Michael Wink

**Affiliations:** Institute of Pharmacy and Molecular Biotechnology, University of Heidelberg, Im Neuenheimer Feld 364, Heidelberg 69120, Germany; E-Mails: thorsten@benz-ettlingen.de (T.B.); c-zapp@t-online.de (C.Z.)

**Keywords:** cytosolic phospholipase A_2_α, anti-inflammatory, arachidonic acid, medicinal plants, phenolic content, antioxidant activity, DPPH, Folin–Ciocalteu reagent

## Abstract

The cytosolic phospholipase A_2_α (cPLA${}_{2}\alpha$) is one of the potential targets for anti-inflammatory drugs, since this enzyme plays a key role in the inflammation processes seen in health disorders, like asthma, allergic reactions, arthritis and neuronal diseases. In this study, cPLA${}_{2}\alpha$ inhibition by 43 methanol extracts from medicinal plants rich in polyphenols was determined. The eight most active extracts were derived from *Ribes nigrum* (IC${}_{50}$ of 27.7 µg/mL), *Ononis spinosa* (IC${}_{50}$ of 39.4 µg/mL), *Urtica dioica* (IC${}_{50}$ of 44.32 µg/mL), *Betula* sp. (IC${}_{50}$ of 58.02 µg/mL), *Sanguisorba officinalis* (IC${}_{50}$ of 76.25 µg/mL), *Orthosiphon stamineus* (IC${}_{50}$ of 78.83 µg/mL), *Petasites hybridus* (IC${}_{50}$ of 81.02 µg/mL) and *Tussilago farfara* (IC${}_{50}$ of 123.28 µg/mL). Additionally, the antioxidant activities of these extracts were determined with the 2,2-diphenyl-1-picrylhydrazyl (DPPH) assay and their phenolic content with the Folin–Ciocalteu reagent. Antioxidant activity showed a non-linear, positive correlation to the phenolic content, but no correlation of PLA${}_{2}\alpha$ inhibition with phenolic content could be established. This study provides evidence that cPLA${}_{2}\alpha$ may be a relevant target for anti-inflammatory agents.

## 1. Introduction

The phospholipases A${}_{2}$ (PLA${}_{2}$) play an important role in the inflammatory response. The enzymes catalyze the release of a fatty acid at the *sn*${}_{2}$-position of membrane phospholipids. This leads to the release of a lysophospholipid, which itself functions as a signal molecule, and arachidonic acid (AA), a poly-unsaturated fatty acid. AA is the precursor for the synthesis of the eicosanoids, which are lipid mediators of the inflammatory response. Two well-known subgroups of eicosanoids are the prostaglandins and leukotrienes, which are produced from AA through the enzymes cyclooxygenase 1 and 2 (COX-1 and COX-2) and 5-lipoxygenase (5-LOX), respectively [1]. Whereas COX enzymes are well-studied targets for anti-inflammatory drugs like acetylsalicylic acid, no drugs are on the market for 5-LOX and PLA${}_{2}$, so far. Depending on their size, catalytic mechanism and specificity for AA, there are more than ten groups of PLA${}_{2}$ enzymes [1]. Among PLA${}_{2}$ enzymes, the cytosolic PLA${}_{2}$ alpha from Subgroup IV (cPLA${}_{2}\alpha$) is known to have the highest specificity for AA [2] and, therefore, is discussed as the key enzyme in the release of AA for eicosanoid synthesis [1,3]. cPLA${}_{2}\alpha$ has been associated with inflammation-related diseases, like asthma and allergic reactions [4,5,6], arthritis [7,8] and neuronal diseases, such as Alzheimer’s disease [9,10], multiple sclerosis [11] and Parkinson’s disease [12]. Therefore, the cPLA${}_{2}\alpha$ isoform should be a potential target for anti-inflammatory drugs [13].

In the history of the therapy of inflammation, medicinal plants have always played important roles [14]; still, their mechanisms of action are often unknown. In this study, porcine cPLA${}_{2}\alpha$ inhibition by 43 methanol extracts of different medicinal plants was analyzed, known to accumulate potentially anti-inflammatory polyphenols, such as flavonoids, which are known to have an influence on the arachidonic acid metabolism [14,15,16]. Following this screening, we further examined the eight most potent inhibitory extracts in more detail. In addition, the total phenolic content of these extracts was quantified with Folin–Ciocalteu reagent and their antioxidant activity with DPPH. We further analyzed whether anti-inflammatory properties are correlated with phenolic content and anti-oxidant activities.

## 2. Results and Discussion

**Figure 1:**
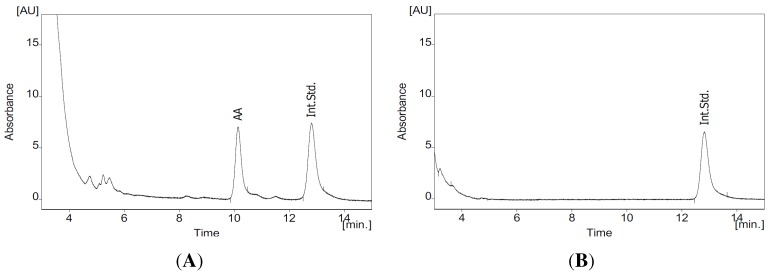
HPLC chromatogram of a sample of *Ononis spinosa* herb extract (100 µg/mL) incubated with cPLA_2_α. Absorbance (AU, y-axis) is depicted in relation to time (min, x-axis) at a detection wavelength of 200 nm (**A**) and 254 nm (**B**), respectively. AA = arachidonic acid peak; Int.Std. = the peak of the internal standard 4-undecyloxybenzoic acid.

Initially, 43 methanol extracts from 43 species of 25 different plant families were screened for cPLA${}_{2}\alpha$ inhibition at a final concentration of 100 µg/mL. As a positive control, the cPLA${}_{2}\alpha$ inhibitor arachidonyl trifluoromethyl ketone (25 µM) was employed, resulting in an 80% inhibition. All experiments were conducted the same way, and no abnormalities occurred. Figure 1 shows an exemplary HPLC chromatogram from a sample of 100 µg/mL *Ononis spinosa* incubated with cPLA${}_{2}\alpha$. Among the active extracts, 17 showed over 50% inhibition of AA release (see Table 1 for all screening results). From these, the eight best inhibitory extracts, *Urtica dioica* (leaf), *Petasites hybridus* (leaf), *Sanguisorba officinalis* (herb), *Ribes nigrum* (leaf), *Betula* sp. (leaf), *Ononis spinosa* (herb), *Orthosiphon stamineus* (leaf) and *Tussilago farfara* (leaf), in descending order, were chosen to establish the dose dependence of cPLA${}_{2}\alpha$ inhibition.

The activity of cPLA${}_{2}\alpha$ strongly depends on calcium ions, which are necessary for its binding to substrate [2,17]. Furthermore, enzymatic activity can be raised two to three times by phosphorylation [18,19]. It has been shown that phosphorylation of human cPLA${}_{2}\alpha$ takes place within five minutes after stimulation of platelets with thrombin [18,20]. In our experimental setup, we isolate the porcine cPLA${}_{2}\alpha$ from fresh blood. In the process of slaughtering, the pigs experience stress and injury, which may trigger the production of thrombin. Thus, during the process of platelet isolation from porcine blood in the lab, phosphorylation of porcine cPLA${}_{2}\alpha$ may take place. However, we did not observe platelet coagulation as a consequence of thrombin production.

**Table 1:** cPLA${}_{2}\alpha$ inhibition (%) by 100 µg/mL methanol extract of 43 plants. Species are ordered according to their inhibitory activity.

Scientific Name	Plant Family	Part	Drug Name	Inhibition ± Error (%)
*Urtica dioica* L.	Urticaceae	leaf	Urticae folium	74.7 ± 2.87
*Petasites hybridus* (L.) Gaertn.	Asteraceae	leaf	Petasitidis folium	73.01 ± 2.42
*Sanguisorba officinalis* L.	Rosaceae	herb	Sanguisorbae herba	70.24 ± 2.88
*Ribes nigrum* L.	Grossulariaceae	leaf	Ribis nigri folium	69.49 ± 8.49
*Betula* sp. L.	Betulaceae	leaf	Betulae folium	68.69 ± 13.3
*Ononis spinosa* L.	Fabaceae	herb	Oninidis herba	67.97 ± 7.94
*Orthosiphon stamineus* Benth	Lamiaceae	leaf	Orthosiphonis folium	65.71 ± 7.64
*Tussilago farfara* L.	Asteraceae	leaf	Farfarae folium	54.91 ± 3.82
*Paullinia cupana* Kunth	Sapindaceae	fruit	Guaranae fructus	54.71 ± 3.86
*Senegalia catechu* (L.F.) Hurter & Mabb.	Fabaceae	gum	Gummi catechu	54.61 ± 7.2
*Styphnolobium japonicum* (L.) Schott	Fabaceae	flower	Sophorae flos	53.68 ± 2.69
*Helichrysum arenarium* (L.) Moench	Asteraceae	flower	Helichrysi flos	53.12 ± 3.34
*Hamamelis virginiana* L.	Hamamelidaceae	leaf	Hamamelidis folium	52.82 ± 5.67
*Camellia sinensis* (L.) Kuntze	Theaceae	leaf	Theae folium	52.46 ± 2.93
*Centella asiatica* (L.) Urban	Apiaceae	herb	Centellae herba	51.85 ± 4.07
*Tropaeolum majus* L.	Tropaeolaceae	herb	Tropaeoli herba	50.85 ± 3.59
*Arnica montana* L.	Asteraceae	flower	Arnicae flos	50.06 ± 4.1
*Tanacetum parthenium* (L.) Sch.Bip.	Asteraceae	herb	Tanaceti parthenii herba	49.46 ± 3.92
*Cynara cardunculus* L.	Asteraceae	leaf	Cynarae folium	44.85 ± 4.52
*Plantago lanceolata* L.	Plantaginaceae	leaf	Plantaginis lanceolatae folium	42.25 ± 3.93
*Leonurus cardiaca* L.	Lamiaceae	herb	Leonuri herba	41.87 ± 3.79
*Marsdenia cundurango* Rchb.f.	Apocynaceae	cortex	Condurango cortex	41.84 ± 5.34
*Melissa officinalis* L.	Lamiaceae	leaf	Melissae folium	41.53 ± 4.48
*Agathosma betulina* (Berg.) Pillans	Rutaceae	leaf	Bucco folium	39.49 ± 5.55
*Solidago* sp. L.	Asteraceae	herb	Solidaginis herba	36.66 ± 5.12
*Quercus* sp. L.	Fagaceae	cortex	Quercus cortex	36.29 ± 3.95
*Salix alba* L.	Salicaceae	cortex	Salicis cortex	34.33 ± 5.71
*Crataegus* sp. Tourn. ex L.	Rosaceae	flower	Crataegi flos	29.83 ± 18.31
*Vaccinium vitis-idaea* L.	Ericaceae	leaf	Vitis idaei folium	29.36 ± 6.88
*Harungana madagascariensis* Lam. ex Poiret	Hypericaceae	cortex	Harongae cortex	29.36 ± 4.16
*Peumus boldus* Molina	Monimiaceae	leaf	boldo folium	28.46 ± 8.37
*Salvia officinalis* L.	Lamiaceae	leaf	Salviae folium	26.16 ± 7.89
*Berberis vulgaris* L.	Berberidaceae	cortex	Berberidis cortex	24.9 ± 4.27
*Juglans regia* L.	Juglandaceae	leaf	Juglandis folium	23.88 ± 4.45
*Arctostaphylos uva-ursi* (L.) Spreng.	Ericaceae	leaf	Uvae-ursi folium	22.04 ± 5.75
*Verbascum* sp. L.	Scrophulariaceae	flower	Verbasci flos	21.64 ± 4.49
*Vaccinium myrtillus* L.	Ericaceae	leaf	Myrtilli folium	19.17 ± 6.68
*Filipendula ulmaria* (L.) Maxim.	Rosaceae	herb	Filipendulae herba	10.19 ± 10.43
*Alchemilla vulgaris* L.	Rosaceae	herb	Alchemillae herba	10.03 ± 5.76
*Fragaria vesca* L.	Rosaceae	leaf	Fragariae folium	7.28 ± 5.14
*Hamamelis virginiana* L.	Hamamelidaceae	cortex	Hamamelidis cortex	1.41 ± 12.42
*Punica granatum* L.	Lythraceae	cortex	Granati cortex	−10.69 ± 6.81
*Humulus lupulus* L.	Cannabaceae	glands	Lupuli glandula	−65.16 ± 10.73
Arachidonyl Trifluoromethyl Ketone (25 µM)	-	-	-	80.04 ± 3.58

### 2.1. Dose Dependence of cPLA${}_{\text{2}}\alpha$ Inhibition

**Figure 2:**
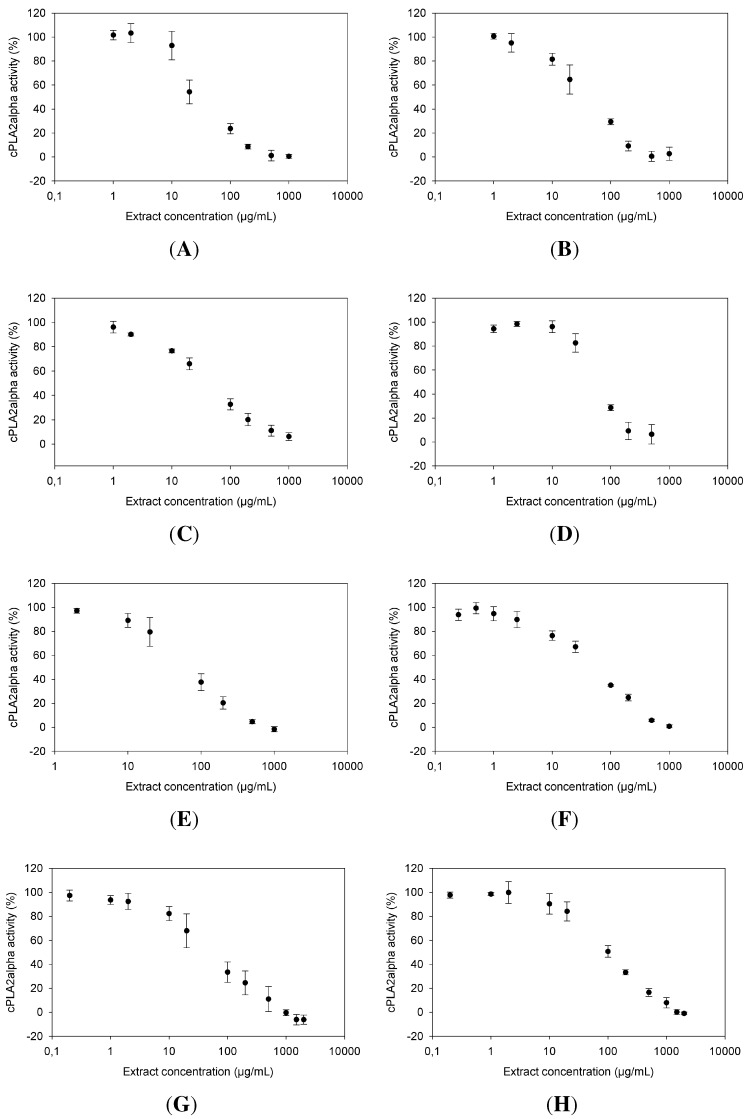
Dose-dependence of cytosolic phospholipase A_2_α (cPLA_2_α) inhibition by extracts of: *Ribes nigrum* (**A**); *Ononis spinosa* (**B**); *Urtica dioica* (**C**); *Betula sp.* (**D**); *Sanguisorba officinalis* (**E**); *Orthosiphon stamineus* (**F**); *Petasites hybridus* (**G**); and *Tussilago farfara* (**H**). cPLA_2_α activity (%) is plotted against extract concentration (µg/mL). The x-axes are scaled logarithmically.

All eight extracts exhibited a dose-dependent cPLA${}_{2}\alpha$ inhibition (Figure 2). With an IC${}_{50}$ value of 27.7 ± 4.71 µg/mL, the extract from *Ribes nigrum* was the most potent cPLA${}_{2}\alpha$ inhibitor (Figure 2A), followed by *Ononis spinosa* (39.4 ± 6.49 µg/mL) (Figure 2B), *Urtica dioica* (44.32 ± 5.88 µg/mL) (Figure 2C) and *Betula* sp. (58.02 ± 5.99 µg/mL) (Figure 2D). A little less active were the extracts of *Sanguisorba officinalis* (Figure 2E), *Orthosiphon stamineus* (Figure 2F) and *Petasites hybridus* (Figure 2G), with corresponding IC${}_{\text{50}}$ values of 76.25 ± 10.93 µg/mL, 78.83 ± 15.55 µg/mL and 81.02 ± 18.23 µg/mL, respectively. The least active extract in this selection came from *Tussilago farfara* with an IC${}_{50}$ of 123.28 ± 15.06 µg/mL (Figure 2H).

Previous studies have reported anti-inflammatory activity for four of the eight plants, *Urtica dioica*, *Orthosiphon stamineus*, *Ribes nigrum* and *Ononis spinosa*, in the carragenan-induced paw edema model in rats [21,22,23,24,25,26]. The inflammatory reaction to carrageenan is biphasic: in the first phase, there is a release of histamine and serotonin, whereas the second phase is characterized by the release of eicosanoids, like prostaglandins [27,28,29]. This model can thus be used to study non-specific inflammation, including the release of arachidonic acid through cPLA${}_{2}\alpha$. In addition to the effect in the paw edema model, *Urtica dioica* extracts were shown to inhibit COX-1 and COX-2 directly and to act as an antagonist at the histamine 1 receptor, a key target for allergic reactions [30,31]. With regard to the role of cPLA${}_{2}\alpha$ enzyme in allergic reactions [4,5,6], the cPLA${}_{2}\alpha$ inhibiting the potential of *Urtica dioica* may be important as an anti-allergy agent.

As concerns *Sanguisorba officinalis* and *Ononis spinosa*, the roots are well-known traditional phytomedicines [14]. For the aerial parts of these plants, anti-inflammatory activities have not been reported. Root extracts of *Sanguisorba officinalis* exhibited an anti-asthmatic and anti-allergic effect in mouse models [32,33]. *In vitro* assays with lipopolysaccharide and interferon gamma stimulated mouse macrophages showed the potential of a *Sanguisorba officinalis* extract to substantially inhibit prostaglandin, nitric oxide (NO) and tumor necrosis factor alpha production [34,35]. *Ononis spinosa* root extract showed an analgesic effect in mice equivalent to the positive control aspirin [36] and was able to reduce carrageenan-induced paw edema in rats [21]. We now provide evidence that cPLA${}_{2}\alpha$ is probably the target for the anti-inflammatory activity of the aerial parts from *Sanguisorba officinalis* and *Ononis spinosa*.

Prostaglandin and leukotriene synthesis were inhibited by extracts from *Petasites hybridus* [37,38]. Furthermore, extracts from this plant are marketed as a prophylactic for the treatment of migraine [39,40]. However, its use in the therapy of allergic rhinitis and asthma has been discussed controversially [41,42,43,44]. In this study, we provide evidence that *Petasites hybridus* directly inhibits cPLA${}_{2}\alpha$, which would also explain the reported inflammatory and anti-allergic activities. The extract of *Tussilago farfara* and its ingredient tussilagon inhibit NO and prostaglandin production in stimulated murine microglia cells and macrophages [35,45,46]. The anti-inflammatory effect of tussilagone may be due to an induction of heme oxygenase-1 [47] and an inhibition of COX-2 and nitric oxide synthase gene expression [45]. The anti-inflammatory activity is moderate, as seen from our results of cPLA${}_{2}\alpha$ inhibition.

In traditional medicine, *Betula sp.* leaves are used to treat inflammation [14]. This may be the result of a variety of flavonoids and triterpenoids present [48,49,50]. *Betula pendula* leaf extract showed an inhibitory effect against corneal inflammation in rats [51], lymphocyte growth and cell division [52]. Our results indicate that the anti-inflammatory effect of *Betula sp.* may involve the inhibition of cPLA${}_{2}\alpha$.

### 2.2. cPLA${}_{\text{2}}\alpha$ Inhibition in Relation to Phenolic Content and Antioxidant Activities

Phenolic secondary metabolites, for example flavonoids, have the ability to unspecifically interact with all kinds of biomolecules, especially proteins. This results, for example, in an inhibition of the catalytic mechanism of enzymes [53]. As a consequence, polyphenols could directly inhibit cPLA${}_{2}\alpha$, which would lead to a lower AA release. As expected, the results of the Folin–Ciocalteu assay confirmed that all eight extracts contain phenolic ingredients (see Table 2). It is very interesting that four of the plants—*Betula* sp., *Orthosiphon stamineus*, *Urtica dioica* and *Ononis spinosa*—are used as diuretics in phytomedicine [14]. The diuretic effect is attributed to the flavonoid content of these plants. Many flavonoids are known for their anti-inflammatory properties [15,49,54].

The Folin–Ciocalteu reagent not only reacts to phenolic compounds, but also to reducing agents [55]. To evaluate this interference, the reducing power of the extracts was tested with the stable free radical DPPH. As a control, the EC${}_{50}$ value of ascorbic acid was determined and was expectedly low with 3.18 ± 0.15 µg/mL [56]. Several previous studies confirmed a correlation between the phenolic content determined with Folin–Ciocalteu reagent and the reducing capacity of different plant extracts [57,58,59,60]. In view of the mechanism, which in both assays is based on electron transfer, this is no surprise [55].

**Table 2:** Summary of cPLA${}_{2}\alpha$ inhibition, phenolic content and DPPH scavenging activity of the eight most potent extracts. The cPLA${}_{2}\alpha$ inhibition is shown as the IC${}_{\text{50}}$ value (µg/mL final extract concentration); phenolic content relates to gallic acid equivalents (GAE) in mg per g extract. The DPPH radical scavenging is shown as EC${}_{\text{50}}$ (µg/mL final extract concentration). Values are means (*n* = 3) ± the standard error (SE), ordered according to their cPLA${}_{2}\alpha$ inhibitory activity.

	cPLA${}_{2}\alpha$ Inhibition	Phenolic Content	Radical Scavenging
Extract	(IC${}_{\text{50}}$)	(GAE)	(EC${}_{\text{50}}$)
	µg/mL ± SE	mg/g ± SE	µg/mL ± SE
*Ribes nigrum*	27.7 ± 4.71	131.25 ± 7.15	13.36 ± 0.6
*Ononis spinosa*	39.4 ± 6.49	20.55 ± 2.56	271.07 ± 13.13
*Urtica dioica*	44.32 ± 5.88	38.26 ± 2.41	90.5 ± 4.01
*Betula* sp.	58.02 ± 5.99	62.59 ± 2.38	27.17 ± 1.26
*Sanguisorba officinalis*	76.25 ± 10.93	116.96 ± 5.89	14.93 ± 0.66
*Orthosiphon stamineus*	78.83 ± 15.55	50.2 ± 0.26	30.33 ± 2.06
*Petasites hybridus*	81.02 ± 18.23	122.61 ± 4.73	14.27 ± 0.76
*Tussilago farfara*	123.28 ± 15.06	122.39 ± 5.46	14.54 ± 0.72

**Figure 3:**
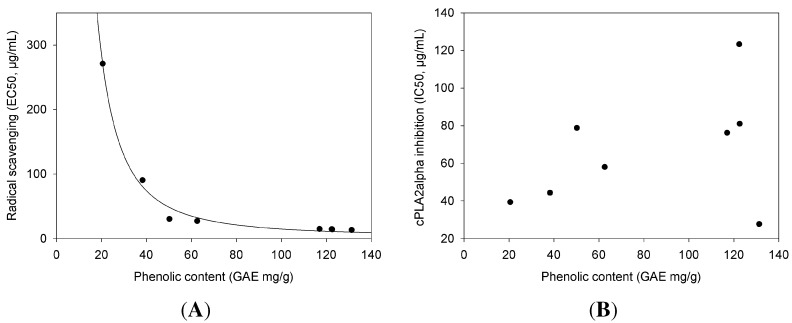
Correlation of antioxidant activities and cPLA_2_α inhibition with the phenolic content of the extracts. In (**A**), the EC values of radical scavenging (µg/mL extract concentration, y-axis) are plotted against the phenolic content (GAE mg/g, x-axis). The non-linear regression curve is calculated with SigmaPlot (polynomial, inverse second order, simplified), resulting in the following equation: $DPPH\;scavenging=3.6+\frac{1.1\cdot 10^{\text{5}}}{{phenolic\;content}^{2}}$; (**B**) The IC values of cPLA_2_α inhibition (µg/mL extract concentration, y-axis) plotted against the phenolic content (GAE mg/g, x-axis). The values do not reveal a correlation. (**A**): DPPH (EC) *vs.* phenolic content; (**B**): cPLA_2_α inh. (IC_50_) *vs.* phenolic content.

EC${}_{\text{50}}$ values for DPPH reduction range between 13.36 ± 0.6 µg/mL (*Ribes nigrum*) and 271.07 ± 13.13 µg/mL (*Ononis spinosa*). With an IC${}_{50}$ value of >200 µg/mL, *Ononis spinosa* may be considered as not active. When our results of the radical scavenging are plotted against the phenolic content, an inverse non-linear correlation can be seen (see Table 2, Columns 2 and 3, and Figure 3A), meaning that the higher the phenolic content, the stronger the antioxidant effects. Hence, the extracts with higher phenolic content also show higher radical scavenging activity (evident in a low EC${}_{\text{50}}$).

While radical scavenging activity and phenolic content correlate very well, cPLA${}_{2}\alpha$ inhibition is apparently not correlated with the phenolic content (Figure 3B). The best example is *Tussilago farfara* extract, which showed the lowest cPLA${}_{2}\alpha$ inhibition by far, combined with the third highest phenolic content. However, *Ribes nigrum* extract showed the highest phenolic content together with the strongest cPLA${}_{2}\alpha$ inhibition (Table 2).

### 2.3. Urtica dioica and Ononis spinosa as Promising New Anti-Inflammatory Drugs with Regard to cPLA${}_{\text{2}}\alpha$ as a Target

Polyphenols unspecifically bind to proteins, thereby often resulting in conformational changes that lead to inhibition of the enzymatic activity [53]. However, phenolic secondary metabolites may not be the main cause for cPLA${}_{2}\alpha$ inhibition, since there seems to be no correlation between phenolic content of the eight methanol extracts and their cPLA${}_{2}\alpha$ inhibition. Furthermore, some polyphenol-rich plant extracts, such as *Punica granatum* and *Humulus lupulus*, did not inhibit cPLA${}_{2}\alpha$, but instead appear to act as agonists, as the AA production was elevated to 110% and 165%, compared to the control (see Table 1). Therefore, other non-phenolic ingredients may be responsible for cPLA${}_{2}\alpha$ inhibition. Two extracts showing very low phenolic content, but high cPLA${}_{2}\alpha$ inhibition, are *Ononis spinosa* and *Urtica dioica*. Since these two extracts seem to be the most promising cPLA${}_{2}\alpha$ inhibitors despite their low phenolic content, we assume that they may contain specific cPLA${}_{2}\alpha$ inhibitors.

## 3. Experimental Section

### 3.1. Reagents and Chemicals

HPLC-grade water and acetonitrile, nordihydroguaiaretic acid (NDGA), 4-undecyloxybenzoic acid, Folin–Ciocalteu phenol reagent (2 N), protease inhibitor cocktail (containing AEBSF, aprotinin, Bestatin, E-64, leupeptin and pepstatin A) were purchased from Sigma-Aldrich (Taufkirchen, Germany); 1-stearoyl-2-arachidonoyl-phosphatidylcholine (SAPC), 1,2-dioleoyl-*sn*-glycerol (DOG) and bromoenol lactone were from Cayman Chemical (Ann Arbor, MI, USA, delivered via Biomol, Hamburg, Germany); dithiothreitol (DTT) from Applichem (Darmstadt, Germany) and ascorbic acid were from Carl Roth (Karlsruhe, Germany).

### 3.2. Isolation of cPLA${}_{\text{2}}\alpha$ from Porcine Platelets

Fresh porcine blood (750 mL) from a nearby slaughterhouse (Fleischversorgungszentrum, Mannheim, Germany) was immediately mixed 1:5 with citrate buffer (85 mM Na${}_{3}$Citrate × 2 H${}_{2}$O, 66.6 mM citric acid and 111 mM D(+)glucose) and subsequently centrifuged at portions of 20 mL (per 50 mL tube) at 2000× *g* (Hettich Rotina 380R, Hettich Lab Technology, Tuttlingen, Germany) for 3 min at 4 ${}^{\circ }$C. The platelet-rich supernatant was transferred to fresh tubes, and platelets were centrifuged at 1600× *g* for 20 min at 4 ${}^{\circ }$C. The pellet was resuspended with 3 mM EDTA in phosphate-buffered saline (137 mM NaCl, 2.68 mM KCl, 10.14 mM Na${}_{2}$HPO${}_{4}$, 1.76 mM KH${}_{2}$PO${}_{4}$, pH 7.5) and centrifuged again. Platelet lysis and cPLA${}_{2}\alpha$ isolation were conducted as described in [61], with minor changes. Briefly, platelets were lysed in a hypotonic buffer at −20 ${}^{\circ }$C over night after the addition of protease inhibitor cocktail. After sonification (Omni-Ruptor 4000, Omni International Inc., Kennesaw, GA, USA, 2 times 30 s, 50% intensity), cell debris was separated by centrifugation at 48,000× *g* for 60 min at 4 ${}^{\circ }$C (Beckman J2-21 centrifuge, rotor type JA-20, Beckman Coulter, Krefeld, Germany). The clear supernatant was diluted 1:1 with Buffer A (25 mM Tris, 1 mM EGTA, 2 mM DTT, pH 8.0) and transferred to a HiPrep™ Q XL 16/10 anionic exchange column (GE Healthcare Europe, Freiburg, Germany, column volume: 20 mL, flow rate: 5 mL/min), which was conditioned with the subsequent addition of 100 mL Buffer A, 100 mL Buffer B (1 M NaCl, 25 mM Tris, 1 mM EGTA, 2 mM DTT, pH 8.0) and 140 mL Buffer A again. After sample loading, the column was washed with 100 mL Buffer A, following a stepwise elution of 4 fractions of 60 mL each. The fractions were eluted with different NaCl concentrations in buffer (realized through a mixture of Buffers A and B), which were 150, 300, 450 and 600 mM each. The 450 mM NaCl fraction contained the cPLA${}_{2}\alpha$ and was concentrated through a centrifugal filter with a molecular weight cut-off of 50 kDa (Vivaspin® 20, Sartorius, Göttingen, Germany) at 4000× *g* (4 ${}^{\circ }$C). The resulting volume of 7 mL was incubated for 10 min at room temperature with bromoenol lactone (5 µM final concentration) to permanently inhibit the activity of the calcium-independent PLA${}_{2}$ isoform. Secreted PLA${}_{2}$ (sPLA${}_{2}$) isoforms have up to eight disulfide bonds essential for their activity. By adding DTT to Buffer A and to the assay buffer, these disulfide bonds get reduced to free thiols, resulting in an activity loss of the sPLA${}_{2}$ isoforms [1,2,62]. In contrast to the sPLA${}_{2}$s, cPLA${}_{2}\alpha$ is only active without disulfide bonds in the reducing environment of the cytosol, which is mimicked by the DTT [62,63]. Other cPLA${}_{2}$ isoforms are considered negligible, since arachidonyl trifluoromethyl ketone, a specific cPLA${}_{2}\alpha$ inhibitor [64], inhibits arachidonic acid production over 96% in our test system (at a concentration of 200 µM). The protein solution was diluted in Buffer A to liberate about 1 nmol AA in 60 min incubation at 37 ${}^{\circ }$C. The enzyme fraction was stored in aliquots at −80 ${}^{\circ }$C.

### 3.3. Incubation Procedure for the cPLA${}_{\text{2}}\alpha$ Assay

The incubation procedure and solid phase extraction was executed as described in [61], with some modifications. In short, 2 µL of extract (or dimethyl sulfoxide (DMSO) in the case of the control and blank) were incubated together with 10 µL isolated enzyme (blank: 10 µL buffer) and 88 µL mixed micelle emulsion as a substrate (formed by 200 µM SAPC and 100 µM DOG). After a 60-min incubation time at 37 ${}^{\circ }$C, the reaction was stopped by adding 200 µL of acetonitrile/methanol/Na${}_{2}$EDTA (16:15:1) containing nordihydroguaiaretic acid (NDGA, 0.6 µg/200 µL) and 4-undecyloxybenzoic acid as the internal standard (156 ng/200 µL). After solid-phase extraction according to [65] with BondElut C18 columns (100 mg sorbent, 3 mL volume; Agilent technologies, Santa Clara, CA, USA), the eluate was diluted 1:1 with water for HPLC analysis.

The initial PLA${}_{2}\alpha$ inhibition screening was conducted with 43 methanol extracts (final concentration: 100 µg/mL), once in duplets. To establish dose dependence, the eight most potent inhibitors were tested in different concentrations in duplets in three independent experiments (*n* = 3). Controls and blanks were carried out in triplets for all experiments.

### 3.4. HPLC Analysis of AA

For quantification of AA, a Young Lin YL9100 HPLC system (Young Lin Instrument Co. Ltd., Anyang, Korea, quaternary pump, degasser and diode array detector) connected to a Spark Marathon autosampler (Spark Holland, Emmen, The Netherlands) with a 200 µL sample loop was used. Using an isocratic program described in [65] with acetonitrile/water/phosphoric acid (77:23:0.1 *v*/*v*/*v*) as the mobile phase, 200 µL of the prepared sample was injected, and chromatographic separation was carried out with a Nucleosil C18 column (Macherey-Nagel, Düren, Germany, 3 mm inner diameter, 3 µm particle size, 125 mm length). AA and internal standard were detected at a wavelengths of 200 nm and 254 nm, respectively. For quantification of the relative enzyme activity, the AA peak area was set in relation to the internal standard peak area. The control (2 µL DMSO, 10 µL enzyme, 88 µL substrate) was defined as 100% activity, whereas the blank (2 µL DMSO, 10 µL buffer and 88 µL substrate) was defined as 0% activity.

### 3.5. Quantification of the Phenolic Agents with Folin–Ciocalteu Reagent

This assay was used to quantify the phenolic content of the extracts. It is an adapted method from [66]. From each extract, 20 µL of a dilution in DMSO (1 mg/mL) was transferred in triplets to a 96-well plate. After addition of the Folin–Ciocalteu reagent (100 µL), the mixture was alkalinized with 80 µL of Na${}_{2}$CO${}_{3}$ solution (7.5% (*w*/*v*) in HPLC-grade water). For calibration, a dilution series of gallic acid (0 to 1.25 mg/mL) was treated the same way. Subsequently, the 96-well plate was incubated in the dark at room temperature for 60 min, following the measurement of absorption at 750 nm with a plate reader (Tecan Infinite M200 Pro, Tecan Group Ltd., Männedorf, Switzerland). To minimize the influence of inherent absorption from the extracts, the absorption of blanks containing 100 µL water instead of Folin–Ciocalteu reagent were subtracted from the absorptions of the samples. The same was done for the gallic acid standards. Using gallic acid, a calibration curve was established, and the phenolic content of the extracts was calculated using gallic acid equivalents (GAE), quantified as mg of gallic acid per g of extract.

### 3.6. DPPH Assay

For evaluation of the reducing potential, the DPPH radical scavenging activity was determined. This assay was originally established in [67]. One hundred microliters of diluted extract were transferred to a 96-well plate in duplicates. Subsequently, 100 µL of DPPH solution (0.2 mM in methanol) were added, and incubation took place for 30 min in the dark at room temperature, following the determination of the absorption at 517 nm (Tecan Infinite M200 Pro). Final concentrations of the extracts ranged between 0.3 µg/mL to 5 mg/mL. As a control, 100 µL methanol was mixed with 100 µL of DPPH solution. The inherent absorption of the extracts was measured separately (extract and methanol) and subtracted from each sample’s absorption. Ascorbic acid (0.06 µg/mL to 62.5 µg/mL) served as a standard control. Radical scavenging was calculated with the following formula:

$$scavenging\left(\%\right)=100\cdot \frac{A_{0}-A_{s}}{A_{0}}$$

with $A_{0}$ = control (DPPH + methanol) and $A_{s}$ = sample (DPPH + extract or ascorbic acid, respectively).

### 3.7. Statistics

For the PLA${}_{2}\alpha$ inhibition screening, errors were calculated with Gaussian error propagation. Standard deviations from the mean values were calculated for the dose-dependent studies and phenolic content. The IC${}_{\text{50}}$ and EC${}_{\text{50}}$ concentrations and their standard errors were calculated with SigmaPlot 11.0 (standard curve analysis with four parameter logistic).

## 4. Conclusions

In this study, we have provided evidence that polyphenol-rich medicinal plants often exert anti-inflammatory activities, which may be due to an inhibition of cPLA${}_{2}\alpha$, which has been rather neglected as a relevant target for anti-inflammatory drugs. In another series of experiments, we have tried to identify the active substances in these extracts (Arnold and Wink, in preparation). However, possible synergistic effects from two or more of the ingredients in the extracts should not be left out of consideration [53].

## References

[1] Dennis E.A., Cao J., Hsu Y.H., Magrioti V., Kokotos G. (2011). Phospholipase A_2_ enzymes: Physical structure, biological function, disease implication, chemical inhibition, and therapeutic intervention. Chem. Rev..

[2] Clark J.D., Lin L.L., Kriz R.W., Ramesha C.S., Sultzman L.A., Lin A.Y., Milona N., Knopf J.L. (1991). A novel arachidonic acid-selective cytosolic PLA_2_ contains a Ca^2+^-dependent translocation domain with homology to PKC and GAP. Cell.

[3] Balsinde J., Winstead M.V., Dennis E.A. (2002). Phospholipase A_2_ regulation of arachidonic acid mobilization. FEBS Lett..

[4] Sokolowska M., Stefanska J., Wodz-Naskiewicz K., Cieslak M., Pawliczak R. (2010). Cytosolic phospholipase A_2_ group IVA is overexpressed in patients with persistent asthma and regulated by the promoter microsatellites. J. Allergy Clin. Immunol..

[5] Mruwat R., Yedgar S., Lavon I., Ariel A., Krimsky M., Shoseyov D. (2013). Phospholipase A_2_ in experimental allergic bronchitis: A lesson from mouse and rat models. PLoS ONE.

[6] Pniewska E., Sokolowska M., Kupryś-Lipińska I., Przybek M., Kuna P., Pawliczak R. (2014). The step further to understand the role of cytosolic phospholipase A_2_ Alpha and group X secretory phospholipase A_2_ in allergic inflammation: Pilot study. Biomed. Res. Int..

[7] Sommerfelt R.M., Feuerherm A.J., Jones K., Johansen B. (2013). Cytosolic phospholipase A2 regulates TNF-induced production of joint destructive effectors in synoviocytes. PLoS ONE.

[8] Kokotos G., Feuerherm A.J., Barbayianni E., Shah I., Sæther M., Magrioti V., Nguyen T., Constantinou-Kokotou V., Dennis E.A., Johansen B. (2014). Inhibition of group IVA cytosolic phospholipase A_2_ by thiazolyl ketones *in vitro*, *ex vivo*, and *in vivo*. J. Med. Chem..

[9] Stephenson D., Rash K., Smalstig B., Roberts E., Johnstone E., Sharp J., Panetta J., Little S., Kramer R., Clemens J. (1999). Cytosolic phospholipase A_2_ is induced in reactive glia following different forms of neurodegeneration. Glia.

[10] Sagy-Bross C., Kasianov K., Solomonov Y., Braiman A., Friedman A., Hadad N., Levy R. (2014). The role of Cytosolic Phospholipase A_2_α in Amyloid Precursor Protein induction by amyloid beta_1–42_-Implication for Neurodegeneration. J. Neurochem..

[11] Kalyvas A., David S. (2004). Cytosolic phospholipase A_2_ plays a key role in the pathogenesis of multiple sclerosis-like disease. Neuron.

[12] Lee H.J., Bazinet R.P., Rapoport S.I., Bhattacharjee A.K. (2010). Brain arachidonic acid cascade enzymes are upregulated in a rat model of unilateral Parkinson disease. Neurochem. Res..

[13] Meyer M.C., Rastogi P., Beckett C.S., McHowat J. (2005). Phospholipase A_2_ inhibitors as potential anti-inflammatory agents. Curr. Pharm. Des..

[14] Van Wyk B.E., Wink M. (2004). Medicinal Plants of the World: An Illustrated Scientific Guide to Important Medicinal Plants and Their Uses.

[15] Serafini M., Peluso I., Raguzzini A. (2010). Flavonoids as anti-inflammatory agents. Proc. Nutr. Soc..

[16] Ferrándiz M.L., Alcaraz M.J. (1991). Anti-inflammatory activity and inhibition of arachidonic acid metabolism by flavonoids. Agents Actions.

[17] Channon J.Y., Leslie C.C. (1990). A calcium-dependent mechanism for associating a soluble arachidonoyl-hydrolyzing phospholipase A_2_ with membrane in the macrophage cell line RAW 264.7. J. Biol. Chem..

[18] Kramer R.M., Roberts E.F., Manetta J.V., Hyslop P.A., Jakubowski J.A. (1993). Thrombin-induced phosphorylation and activation of Ca^2+^-sensitive cytosolic phospholipase A_2_ in human platelets. J. Biol. Chem..

[19] Lin L.L., Wartmann M., Lin A.Y., Knopf J.L., Seth A., Davis R.J. (1993). cPLA_2_ is phosphorylated and activated by MAP kinase. Cell.

[20] Kramer R.M., Roberts E.F., Strifler B.A., Johnstone E.M. (1995). Thrombin induces activation of p38 MAP kinase in human platelets. J. Biol. Chem..

[21] Bolle P., Faccendini P., Bello U., Panzironi C., Tita B. (1993). *Ononis spinosa* L.: Pharmacological effect of ethanol extract. Pharmacol. Res..

[22] Garbacki N., Tits M., Angenot L., Damas J. (2004). Inhibitory effects of proanthocyanidins from *Ribes nigrum* leaves on carrageenin acute inflammatory reactions induced in rats. BMC Pharmacol..

[23] Dar S.A., Ganai F.A., Yousuf A.R., Balkhi M.U.H., Bhat T.M., Sharma P. (2013). Pharmacological and toxicological evaluation of *Urtica dioica*. Pharm. Biol..

[24] Declume C. (1989). Anti-inflammatory evaluation of a hydroalcoholic extract of black currant leaves (*Ribes nigrum*). J. Ethnopharmacol..

[25] Hajhashemi V., Klooshani V. (2013). Antinociceptive and anti-inflammatory effects of *Urtica dioica* leaf extract in animal models. Avicenna J. Phytomedicine.

[26] Yam M.F., Lim V., Salman I.M., Ameer O.Z., Ang L.F., Rosidah N., Abdulkarim M.F., Abdullah G.Z., Basir R., Sadikun A. (2010). HPLC and anti-inflammatory studies of the flavonoid rich chloroform extract fraction of *Orthosiphon stamineus* leaves. Molecules.

[27] Di Rosa M., Giroud J.P., Willoughby D.A. (1971). Studies on the mediators of the acute inflammatory response induced in rats in different sites by carrageenan and turpentine. J. Pathol..

[28] Di Rosa M. (1972). Biological properties of carrageenan. J. Pharm. Pharmacol..

[29] Vinegar R., Truax J.F., Selph J.L., Johnston P.R., Venable A.L., McKenzie K.K. (1987). Pathway to carrageenan-induced inflammation in the hind limb of the rat. Fed. Proc..

[30] Farag M.A., Weigend M., Luebert F., Brokamp G., Wessjohann L.A. (2013). Phytochemical, phylogenetic, and anti-inflammatory evaluation of 43 *Urtica* accessions (stinging nettle) based on UPLC-Q-TOF-MS metabolomic profiles. Phytochemistry.

[31] Roschek B., Fink R.C., McMichael M., Alberte R.S. (2009). Nettle extract (*Urtica dioica*) affects key receptors and enzymes associated with allergic rhinitis. Phytother. Res..

[32] Lee N.H., Lee M.Y., Lee J.A., Jung D.Y., Seo C.S., Kim J.H., Shin H.K. (2010). Anti-asthmatic effect of *Sanguisorba officinalis* L. and potential role of heme oxygenase-1 in an ovalbumin-induced murine asthma model. Int. J. Mol. Med..

[33] Shin T.Y., Lee K.B., Kim S.H. (2002). Anti-allergic effects of Sanguisorba officinalis on animal models of allergic reactions. Immunopharmacol. Immunotoxicol..

[34] Yu T., Lee Y.J., Yang H.M., Han S., Kim J.H., Lee Y., Kim C., Han M.H., Kim M.Y., Lee J. (2011). Inhibitory effect of *Sanguisorba officinalis* ethanol extract on NO and PGE_2_ production is mediated by suppression of NF-κB and AP-1 activation signaling cascade. J. Ethnopharmacol..

[35] Ravipati A.S., Zhang L., Koyyalamudi S.R., Jeong S.C., Reddy N., Bartlett J., Smith P.T., Shanmugam K., Münch G., Wu M.J. (2012). Antioxidant and anti-inflammatory activities of selected Chinese medicinal plants and their relation with antioxidant content. BMC Complement. Altern. Med..

[36] Yılmaz B.S., Ozbek H., Citoğlu G.S., Uğraş S., Bayram I., Erdoğan E. (2006). Analgesic and hepatotoxic effects of *Ononis spinosa* L.. Phytother. Res..

[37] Fiebich B.L., Grozdeva M., Hess S., Hüll M., Danesch U., Bodensieck A., Bauer R. (2005). *Petasites hybridus* extracts *in vitro* inhibit COX-2 and PGE_2_ release by direct interaction with the enzyme and by preventing p42/44 MAP kinase activation in rat primary microglial cells. Planta Med..

[38] Bickel D., Röder T., Bestmann H.J., Brune K. (1994). Identification and characterization of inhibitors of peptido-leukotriene-synthesis from *Petasites hybridus*. Planta Med..

[39] Agosti R., Duke R.K., Chrubasik J.E., Chrubasik S. (2006). Effectiveness of *Petasites hybridus* preparations in the prophylaxis of migraine: A systematic review. Phytomedicine.

[40] Lipton R.B., Göbel H., Einhäupl K.M., Wilks K., Mauskop A. (2004). *Petasites hybridus* root (butterbur) is an effective preventive treatment for migraine. Neurology.

[41] Käufeler R., Polasek W., Brattström A., Koetter U. (2006). Efficacy and safety of butterbur herbal extract Ze 339 in seasonal allergic rhinitis: Postmarketing surveillance study. Adv. Ther..

[42] Grossmann M., Schmidramsl H. (2000). An extract of *Petasites hybridus* is effective in the prophylaxis of migraine. Int. J. Clin. Pharmacol. Ther..

[43] Thomet O.A.R., Simon H.U. (2002). Petasins in the treatment of allergic diseases: Results of preclinical and clinical studies. Int. Arch. Allergy Immunol..

[44] Guo R., Pittler M.H., Ernst E. (2007). Herbal medicines for the treatment of allergic rhinitis: A systematic review. Ann. Allergy Asthma Immunol..

[45] Lim H.J., Lee H.S., Ryu J.H. (2008). Suppression of inducible nitric oxide synthase and cyclooxygenase-2 expression by tussilagone from Farfarae flos in BV-2 microglial cells. Arch. Pharm. Res..

[46] Qin Z.B., Zhang J., Wu X.D., He J., Ding L.F., Peng L.Y., Li X.Y., Li Y., Deng L., Guo Y.D. (2014). Sesquiterpenoids from *Tussilago farfara* and their inhibitory effects on nitric oxide production. Planta Med..

[47] Hwangbo C., Lee H.S., Park J., Choe J., Lee J.H. (2009). The anti-inflammatory effect of tussilagone, from *Tussilago farfara*, is mediated by the induction of heme oxygenase-1 in murine macrophages. Int. Immunopharmacol..

[48] Keinänen M., Julkunen-Tiitto R. (1998). High-performance liquid chromatographic determination of flavonoids in *Betula pendula* and *Betula pubescens* leaves. J. Chromatogr. A.

[49] Guardia T., Rotelli A.E., Juarez A.O., Pelzer L.E. (2001). Anti-inflammatory properties of plant flavonoids. Effects of rutin, quercetin and hesperidin on adjuvant arthritis in rat. Farmaco.

[50] Germanò M.P., Cacciola F., Donato P., Dugo P., Certo G., D’Angelo V., Mondello L., Rapisarda A. (2012). *Betula pendula* leaves: Polyphenolic characterization and potential innovative use in skin whitening products. Fitoterapia.

[51] Wacker K., Gründemann C., Kern Y., Bredow L., Huber R., Reinhard T., Schwartzkopff J. (2012). Inhibition of corneal inflammation following keratoplasty by birch leaf extract. Exp. Eye Res..

[52] Gründemann C., Gruber C.W., Hertrampf A., Zehl M., Kopp B., Huber R. (2011). An aqueous birch leaf extract of *Betula pendula* inhibits the growth and cell division of inflammatory lymphocytes. J. Ethnopharmacol..

[53] Wink M. (2008). Evolutionary advantage and molecular modes of action of multi-component mixtures used in phytomedicine. Curr. Drug Metab..

[54] Mamani Matsuda M., Kauss T., Al Kharrat A., Rambert J., Fawaz F., Thiolat D., Moynet D., Coves S., Malvy D., Mossalayi M.D. (2006). Therapeutic and preventive properties of quercetin in experimental arthritis correlate with decreased macrophage inflammatory mediators. Biochem. Pharmacol..

[55] Huang D., Ou B., Prior R.L. (2005). The chemistry behind antioxidant capacity assays. J. Agric. Food Chem..

[56] Kukić J., Popović V., Petrović S., Mucaji P., Ćirić A., Stojković D., Soković M. (2008). Antioxidant and antimicrobial activity of *Cynara cardunculus* extracts. Food Chem..

[57] Katsube T., Tabata H., Ohta Y., Yamasaki Y., Anuurad E., Shiwaku K., Yamane Y. (2004). Screening for antioxidant activity in edible plant products: Comparison of low-density lipoprotein oxidation assay, DPPH radical scavenging assay, and Folin-Ciocalteu assay. J. Agric. Food Chem..

[58] Cai Y., Luo Q., Sun M., Corke H. (2004). Antioxidant activity and phenolic compounds of 112 traditional Chinese medicinal plants associated with anticancer. Life Sci..

[59] Wojdyło A., Oszmiański J., Czemerys R. (2007). Antioxidant activity and phenolic compounds in 32 selected herbs. Food Chem..

[60] Djeridane A., Hamdi A., Bensania W., Cheifa K., Lakhdari I., Yousfi M. (2013). The *in vitro* evaluation of antioxidative activity, α-glucosidase and α-amylase enzyme inhibitory of natural phenolic extracts. Diabetes Metab. Syndr..

[61] Hanekamp W., Lehr M. (2012). Determination of arachidonic acid by on-line solid-phase extraction HPLC with UV detection for screening of cytosolic phospholipase A_2_α inhibitors. J. Chromatogr. B Anal. Technol. Biomed. Life Sci..

[62] Li B., Copp L., Castelhano A.L., Feng R., Stahl M., Yuan Z., Krantz A. (1994). Inactivation of a cytosolic phospholipase A_2_ by thiol-modifying reagents: cysteine residues as potential targets of phospholipase A_2_. Biochemistry.

[63] Cleland W.W. (1964). Dithiothreitol, a new protective reagent for SH groups. Biochemistry.

[64] Riendeau D., Guay J., Weech P.K., Laliberté F., Yergey J., Li C., Desmarais S., Perrier H., Liu S., Nicoll Griffith D. (1994). Arachidonyl trifluoromethyl ketone, a potent inhibitor of 85-kDa phospholipase A_2_, blocks production of arachidonate and 12-hydroxyeicosatetraenoic acid by calcium ionophore-challenged platelets. J. Biol. Chem..

[65] Schmitt M., Lehr M. (2004). HPLC assay with UV spectrometric detection for the evaluation of inhibitors of cytosolic phospholipase A_2_. J. Pharm. Biomed. Anal..

[66] Singleton V.L., Orthofer R., Lamuela-Raventós R.M. (1999). Analysis of total phenols and other oxidation substrates and antioxidants by means of Folin-Ciocalteu reagent. Methods Enzymol..

[67] Blois M. (1958). Antioxidant determinations by the use of a stable free radical. Nature.

